# Spatial Extent of Charge Repulsion Regulates Assembly Pathways for Lysozyme Amyloid Fibrils

**DOI:** 10.1371/journal.pone.0018171

**Published:** 2011-04-05

**Authors:** Shannon E. Hill, Tatiana Miti, Tyson Richmond, Martin Muschol

**Affiliations:** Department of Physics, University of South Florida, Tampa, Florida, United States of America; University of Naples, Italy

## Abstract

Formation of large protein fibrils with a characteristic cross β-sheet architecture is the key indicator for a wide variety of systemic and neurodegenerative amyloid diseases. Recent experiments have strongly implicated oligomeric intermediates, transiently formed during fibril assembly, as critical contributors to cellular toxicity in amyloid diseases. At the same time, amyloid fibril assembly can proceed along different assembly pathways that might or might not involve such oligomeric intermediates. Elucidating the mechanisms that determine whether fibril formation proceeds along non-oligomeric or oligomeric pathways, therefore, is important not just for understanding amyloid fibril assembly at the molecular level but also for developing new targets for intervening with fibril formation. We have investigated fibril formation by hen egg white lysozyme, an enzyme for which human variants underlie non-neuropathic amyloidosis. Using a combination of static and dynamic light scattering, atomic force microscopy and circular dichroism, we find that amyloidogenic lysozyme monomers switch between three different assembly pathways: from monomeric to oligomeric fibril assembly and, eventually, disordered precipitation as the ionic strength of the solution increases. Fibril assembly only occurred under conditions of net repulsion among the amyloidogenic monomers while net attraction caused precipitation. The transition from monomeric to oligomeric fibril assembly, in turn, occurred as salt-mediated charge screening reduced repulsion among individual charged residues on the same monomer. We suggest a model of amyloid fibril formation in which repulsive charge interactions are a prerequisite for ordered fibril assembly. Furthermore, the spatial extent of non-specific charge screening selects between monomeric and oligomeric assembly pathways by affecting which subset of denatured states can form suitable intermolecular bonds and by altering the energetic and entropic requirements for the initial intermediates emerging along the monomeric *vs.* oligomeric assembly path.

## Introduction

Deposits of insoluble protein fibrils with cross β-sheet structure are the molecular hallmark of an increasing number of human disorders, including Alzheimer's disease, Parkinson's diseases and type II diabetes [1], [2], [3], [4], [5]. Oligomeric intermediates, transiently formed during fibril assembly, are consistently implicated as main culprits responsible for cellular toxicity of both neuropathetic and systemic forms of amyloidoses [6], [7], [8]. At the same time, amyloid polymerization can proceed along multiple assembly pathways, not all of which give rise to oligomeric intermediates [9], [10], [11], [12]. Fibril assembly of β_2_-microglobulin, for example, can yield "worm-like", "rod-like" or "long straight" intermediates [12]. Different aggregation intermediates during amyloid polymerization have been documented, as well, for amyloid-β [9], [11], human serum albumin [13] or the yeast prion Sup-35 NM [14]. Given the significance of oligomeric intermediates to amyloid toxicity, it is important to elucidate the protein and solution attributes regulating fibril assembly pathways. Exposing these mechanisms will not only improve our basic understanding of amyloid fibril self assembly but could help devise new treatment strategies by directing amyloid formation towards non-toxic assembly pathways.

The wide variety of structurally and functionally unrelated proteins that can condense into amyloid fibrils with a common β-sheet structure has led to the suggestion that amyloid fibril formation might be driven more by the generic physical chemistry of polypeptide chains than the specific biochemical and structural details of amyloid proteins [15]. However, it is not clear yet what the relevant biophysical parameters might be. Research on phase separation during protein crystallization suggests one such possible parameter: the "potential of net force". This “potential of net force” represents the average of all (repulsive and attractive) intermolecular interactions among the monomers while they are rapidly tumbling in solution. Solution conditions favorable for protein crystal growth are characterized by a narrow range of negative values, indicating weak net attraction, prevailing under these conditions [16], [17], [18], [19], [20]. Hence, we wondered whether changes in fibril assembly pathways could be correlated to the potential of net force. Yet, we are not aware of any systematic efforts at evaluating the prevailing intermolecular interactions among amyloidogenic proteins under conditions leading to amyloid fibril growth. We were also intrigued by the observation that many *in vitro* assays of fibril formation with native proteins involved highly acidic solution conditions. Under these conditions many proteins become highly charged. For example, lysozyme monomers carry a positive net charge which can reach +15e at pH = 2.0 [21], [22]. Hence, we wanted to determine what role non-specific charge interactions among the partially denatured monomers played during the assembly of amyloid fibrils.

To investigate these questions, we studied *in vitro* amyloid fibril formation by the small enzyme hen egg white lysozyme. There are multiple reasons why lysozyme is a favorable system to investigate these correlations. Human mutants of lysozyme are among the growing class of natively folded proteins implicated in organ-specific or systemic forms of amyloidoses [23], [24]. The disease-related lysozyme mutants are structurally nearly identical to their native counterpart while their thermal stability is reduced [25]. Finally, amyloid fibrils formed by disease mutants are morphologically as well as structurally indistinguishable from those formed by native lysozyme under a wide variety of solution conditions [26]. Hence, results obtained with native lysozyme are likely to be directly applicable to their disease-related mutants. We investigated lysozyme fibril formation at pH = 2.0 and T = 50°C [27], [28], [29]. Under these conditions, dynamic light scattering (DLS) and atomic force microscopy (AFM) can detect and resolve all intermediates along different aggregation pathways while assembly proceeds at a sufficiently fast rate to perform the numerous experiments required for this study.

We characterized the nucleation and growth kinetics of lysozyme fibrils under conditions of increasing charge screening (increasing salt concentrations) using DLS and correlated AFM [28]. Characterizing the morphologies and physical dimensions of intermediates emerging during the assembly process permitted us to distinguish among different assembly pathways. Using CD spectroscopy we monitored whether changes in observed assembly pathways were related to modifications in the residual structure of the denatured protein or to shifts in denaturation temperature. The character and strength of the net intermolecular interactions among the lysozyme monomers under our growth conditions was quantified using static light scattering (SLS).

## Results

### Lysozyme Fibril Growth at Low Salt Concentrations: Self-assembly via Monomeric Filaments

**Figure 1:**
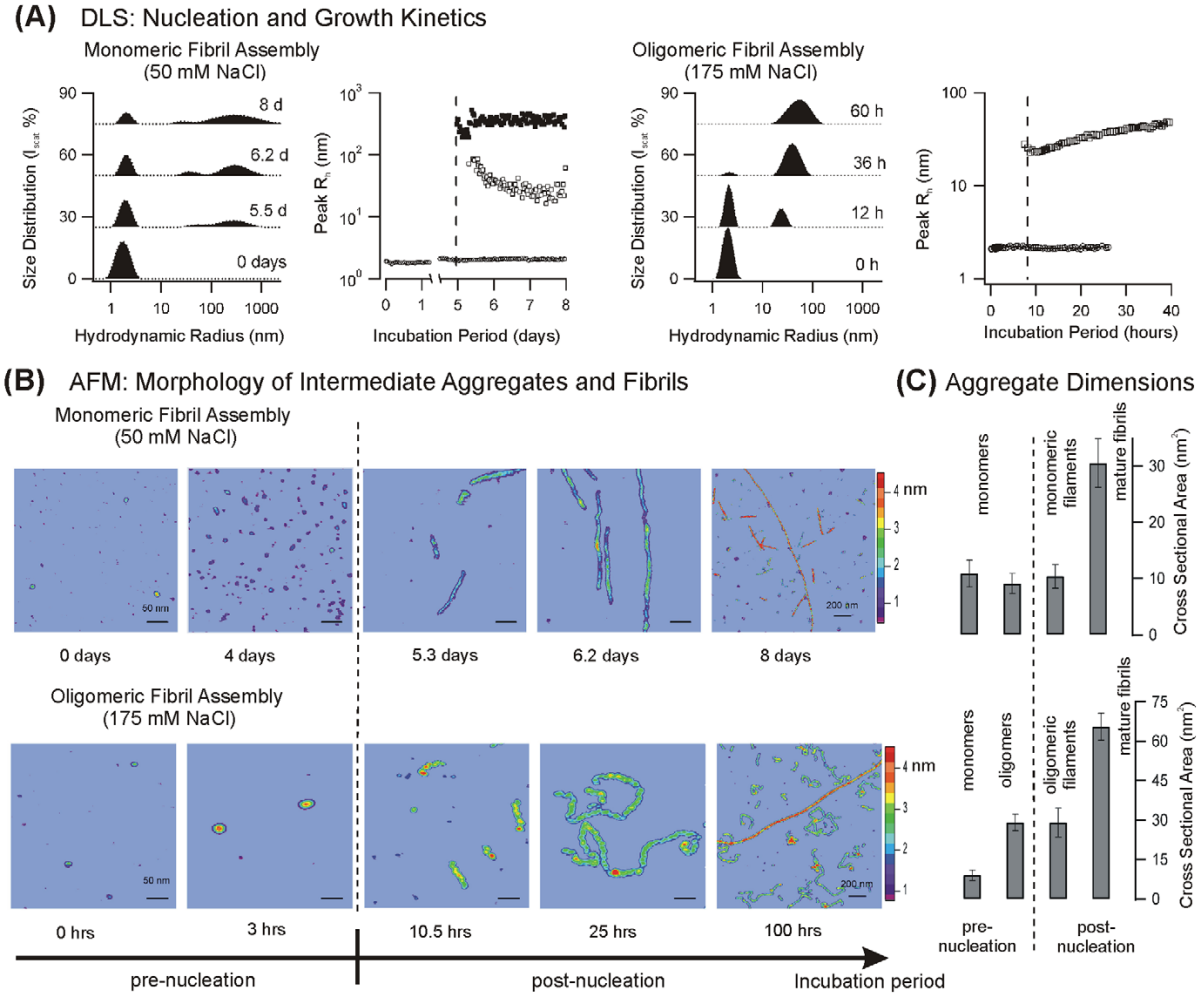
Monomeric vs. Oligomeric Assembly Pathways for Lysozyme Amyloid Fibrils. (**A**) *In situ* particle size distributions at different stages of growth and corresponding temporal evolution of the detected aggregate peaks during lysozyme fibril growth at 50 mM NaCl (left two panels) *vs.* 175 mM NaCl (right two panels), as obtained from dynamic light scattering measurements. The temporal evolution of the aggregate peak radii (1A panel 3&4) highlights the dramatic difference in lag periods (see vertical dashed line) and distinctly different nucleation signatures: Low-salt samples always yielded two peaks while only a single peak nucleated at elevated salt concentrations (**B**) Morphology of growth intermediates in the presence of 50 mM NaCl (top row) *vs.* 175 mM NaCl (bottom row), as observed with atomic force microscopy. The vertical dashed line separates samples taken before and after the nucleation event detected by DLS. The false color scale indicates the height of the different aggregates in nm. The scale bars represent 50 nm, except for the 200 nm scale bars in the last image in either series. AFM images and aggregate dimensions for the 175 mM data are adapted from our earlier work (Hill et al, 2009). They are representative of the behavior observed throughout the "intermediate" salt concentrations (150 mM to 350 mM) associated with the oligomeric assembly regime. (**C**) Cross sectional areas for the various aggregates in (B) measured with calibrated AFM tips. Note the distinctly different cross sections for aggregates along the two different assembly pathways. *Top*: Cross-sectional areas of monomers, monomeric filaments and mature lysozyme fibrils grown at 50 mM NaCl. At low salt, no globular oligomeric species are detected. The cross sections for monomers and monomeric filaments are identical then increase by a factor of three for mature fibrils. *Bottom*: At intermediate salt concentrations, ellipsoidal oligomers are formed well before the nucleation event seen in DLS. These oligomers have a volume close to eight monomers (see Table 1). The filaments emerging after nucleation have a cross section identical to that of the ellipsoidal oligomers. Late stage mature fibrils, in turn, had cross sectional areas close to two oligomeric filaments.

We determined the nucleation and growth kinetics of lysozyme amyloid fibrils grown at different concentrations of sodium chloride by combining DLS with correlated AFM [28]. Below 150 mM fibril assembly kinetics was characterized by extended lag periods lasting multiple days (Fig. 1A, left two panels). Throughout the lag period, only particles with physical dimensions and overall volumes matching monomeric lysozyme were detected in AFM microscopy (Fig. 1B and C, top row and Table 1). During this pre-nucleation period, the shapes of monomers observed with AFM imaging became slightly more elongated and flattened, and their apparent affinity for the mica surface increased (Fig. 1B, top, two left panels). Yet, their overall volume clearly identified them as lysozyme monomers (Table 1). After multiple days of incubation, a nucleation event resulted in the near-simultaneous emergence of not one but *two* aggregate populations with distinctly different hydrodynamic radii. The new aggregate peaks were centered at hydrodynamic radii of around 30 and 300 nm, respectively (Fig. 1A, second panel). AFM images of aliquots removed during DLS measurements indicated that the nucleation event corresponded to the formation of stiff, rod-like polymers (Fig. 1B, top row, panel 3&4).

**Table 1:** Summary of Aggregate Dimensions.

Monomeric Fibril Assembly	Height (nm)	Width (nm)	Cross-section (nm^2^)	Volume (nm^3^)
Monomer (init.)	2.9±0.4	*4.8*±*0.8*/*3.1*±*0.8 ^(^* * *^)^*	10.9±2.4	22.6±7.7
Monomer (pre-nucl.)	2.1±0.4	*5.5*±*0.9*/*4.0*±*1.0 ^(^* * *^)^*	9.1±1.8	24.2±8.5
Monomeric Filaments	2.4±0.4	5.5±0.6	10.4±2.1	
Mature Fibril	5.4±0.3	7.2±0.9	30.5±4.3	

Oligomeric Fibril Assembly	Height (nm)	Width (nm)	Cross-section (nm^2^)	Volume (nm^3^)
Monomer	3.0±0.2 (#)	3.8±0.8 (#)	9.0±2.0	22.7±7.0
Oligomer	3.9±0.1	9.5±1.0	29.1±3.2	184±27.6
Oligomeric Filament	3.9±0.3	9.5±1.7	29.1±5.5	
Mature Fibril	6.0±0.2	13.9±1.0	65.5±5.2	

(*) values quoted for width observed parallel/perpendicular to AFM scan direction.

(#) heights/widths for oligomer pathway match those in our earlier work [27].

The two polymeric filament species emerging after nucleation had cross-sectional areas that were indistinguishable from one another and from the monomers present during the latency period (Table 1). Therefore, we labeled these two populations of nucleating polymers short and long monomeric filaments (MF-S and MF-L), respectively. At the late stages of the incubation period, a third population of thicker fibers appeared in the AFM images without leaving a distinct signature in the DLS signals. The cross-sectional area of these mature fibrils was close to three times that of the monomeric filaments (Fig. 1C, top panel). This suggests that mature fibrils were assembled via intermolecular crosslinking of three monomeric filaments. Overall, the above observations indicate that, at low salt concentrations, amyloid fibril assembly involves the near-simultaneous nucleation of two filament populations of different length. Their cross-sections identified them as linear assemblies of monomers. These monomeric filaments eventually further cross-assembled into thicker, mature fibrils composed of three filaments.

**Figure 2:**
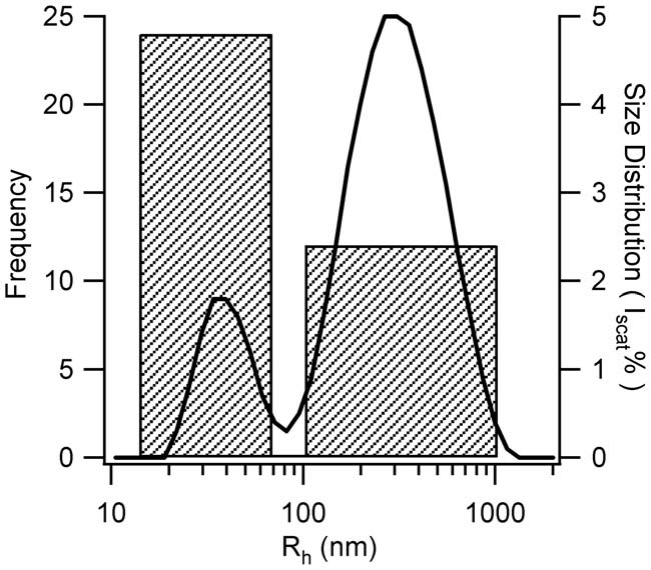
Distribution of Hydrodynamic Radii for Straight Monomeric Filaments: AFM *vs.* DLS. Comparison of the hydrodynamic radii distributions obtained with AFM (shaded bins) *vs*. DLS (solid lines). For ease of comparison, filament lengths measured with AFM were converted into their corresponding hydrodynamic radii using established theoretical predictions for straight cylinders of variable length [30], [31] and diameters close to monomeric filaments (4 nm) or mature fibrils (7 nm).

We further investigated the near-simultaneous nucleation of two aggregate species indicated by DLS. The inversion of DLS correlation functions into aggregate peaks can sometimes introduce spurious peaks. However, analysis of the length distributions for these polymers seen in AFM also yielded a bimodal peak distribution. For a more quantitative comparison of AFM with DLS results we further determined the hydrodynamic radii corresponding to the near-cylindrical filaments seen in AFM (Fig. 1B) using established theoretical predictions [30], [31]. Overall, both DLS and AFM do indicate two distinct aggregate peaks with comparable ranges of hydrodynamic radii (Fig. 2). The noticeable difference in the relative peak amplitudes for short vs. long filaments derived from DLS *vs.* AFM is consistent with the dramatic increase in the sensitivity of DLS for larger aggregates and differences in surface affinity for short vs. long fibers to the mica surfaces used for AFM imaging. Finally, the near-constant values for the hydrodynamic radii *vs*. time for the polymer peaks (Fig. 1A, panel 2) deserve comment. They might result from intrinsic slow growth combined with the insensitivity of the hydrodynamic radius to increases in cylinder length. We prefer the interpretation, instead, that these intermediates exist in a narrow size range, with growth not proceeding *via* monomer addition but *via* assembly of these preformed “building blocks” into higher-order structures.

### Lysozyme Fibril Growth at Intermediate Salt Concentrations: Self Assembly via Oligomeric Intermediates

A distinctly different nucleation and growth pattern was observed at salt concentrations between 150 mM and 350 mM. Lag periods prior to nucleation were shortened from days to a few hours (Fig. 1A, two right panels). DLS measurements still yielded a prominent nucleation event, but only a single new aggregate peak emerged. The initial hydrodynamic radius of these nuclei was around 20–30 nm and grew steadily to about 50 nm within the following 24 hours. The overall width of the nucleated aggregate distribution increased in unison with the hydrodynamic radius. AFM analysis of aggregate morphologies in this regime indicated an assembly pathway distinctly different from that observed at low salt concentrations (Fig. 1B and C, bottom row). Even during the lag period, AFM images revealed the formation of compact oligomeric intermediates. These oligomers had the shape of oblate ellipsoids and were characterized by a tight distribution of physical dimensions [28]. Based on the ellipsoidal oligomer geometry, the oligomer volume was estimated at eight monomers (Table 1). Following nucleation, short polymeric aggregates with a characteristic "beaded" structure emerged (Fig. 1 B). The cross sections of the beaded polymers matched those of the oligomers present prior to nucleation (Table 1). We therefore labeled these polymers oligomeric filaments (OF). Such beaded structures are similarly referred to as protofibrils [12], [32]. As growth proceeded, oligomeric filaments assumed an increasingly curvilinear geometry, again consistent with observations in several other amyloid systems. Near the end of our incubation period, AFM images indicated the formation of larger and much stiffer fibrils. No discernable signature of this late stage event was present in the DLS data. The cross sections for these late-stage fibrils, obtained from calibrated AFM images, were close to twice the cross-sectional area of the oligomeric filaments (table 1). This suggests that oligomeric filaments cross-assembled into double-stranded mature fibrils.

### Aggregation at High Salt Concentrations: Onset of Disordered Precipitation

**Figure 3:**
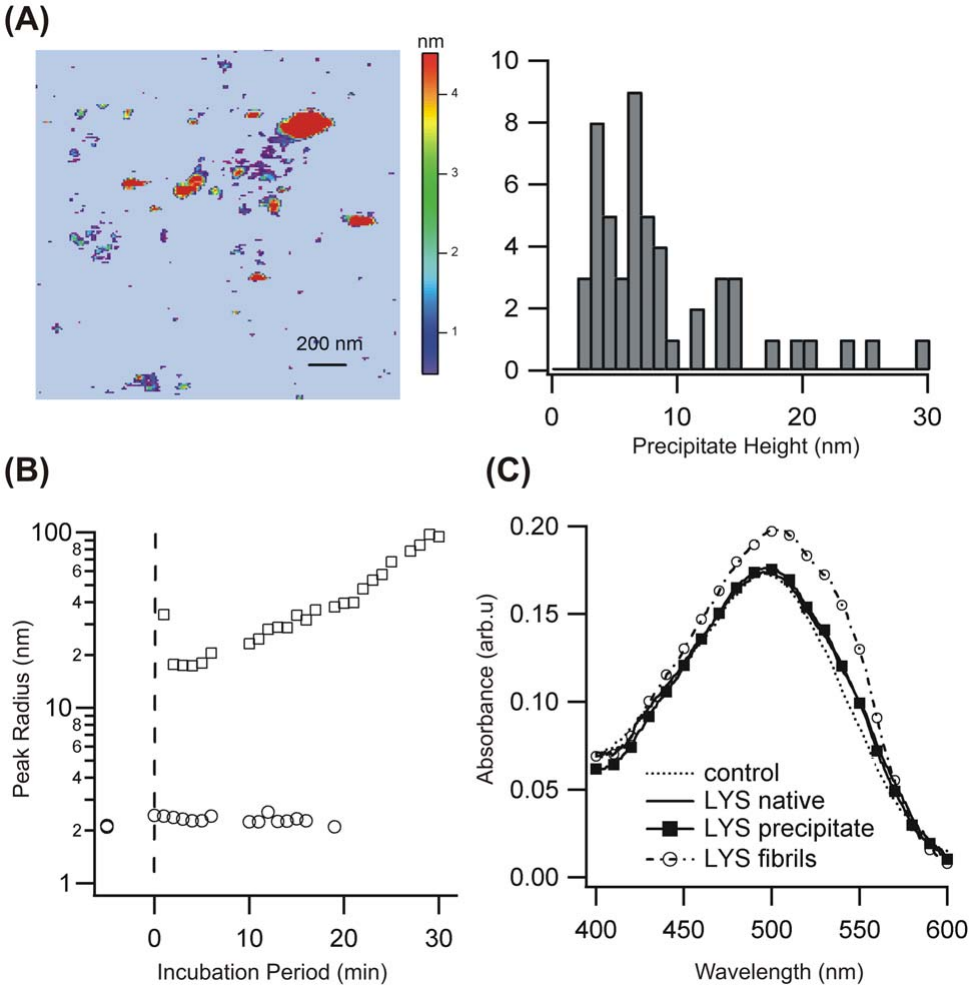
Precipitate Formation of Amyloidogenic Lysozyme. (**A**) AFM image of precipitates and their corresponding height distributions observed shortly after the onset of aggregation. (**B**) DLS aggregate peaks of lysozyme in 400 mM NaCl before and right after partial denaturation of lysozyme (see vertical dashed line). (**C**) Congo Red spectra of native lysozyme (—) and lysozyme precipitates (▪) are indistinguishable. In contrast, mature fibrils grown at lower salt concentrations (open circles) induce the red shift and shoulder characteristic for binding to amyloid fibrils.

With NaCl concentration raised beyond 350 mM, the lag period disappeared completely and a large aggregate peak emerged immediately (Fig. 3B). The scattering intensity associated with these samples increased without any noticeable delay, as well (data not shown). The onset of multiple scattering and inner filtering associated with the rapid growth of aggregates prevented further quantitative analysis of the DLS data. AFM imaging revealed the presence of compact, randomly shaped aggregates of widely different sizes (Fig. 3A). A Congo-Red binding assay indicated that these aggregates did not contain any fibrillar structures. This is in marked contrast to the noticeable enhancement and red-shift seen with samples grown at lower salt concentrations (Fig. 3C). Hence, lysozyme aggregation switched from fibril growth with well-defined populations of intermediates to a precipitation pathway producing a wide distribution of compact precipitates without discernable order or internal structure.

### Structure and Thermal Stability of Lysozyme Monomers

**Figure 4:**
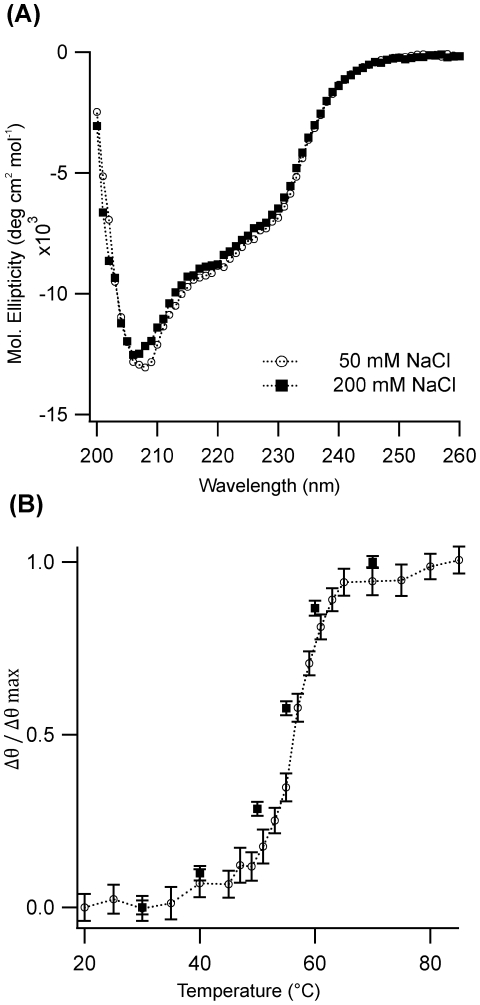
CD Spectroscopy and Thermal Denaturation of Lysozyme Monomers. (**A**) Far uv CD spectra and (**B**) normalized thermal denaturation profile of lysozyme measured at 222 nm in either 50 mM (○) or 200 mM NaCl (▪).

One possible cause for the observed transition in aggregation behavior between low and intermediate salt concentrations could be a salt-induced transition in the structure of the lysozyme monomers or a salt-induced shift in the thermal stability of the monomers. Studies on human lysozyme indicated that the structure of disease-related mutants was only slightly more disordered than those of native lysozyme. However, the stability of the mutants towards thermal denaturation was significantly reduced [24], [25]. We investigated the structure of lysozyme monomers under our partially denaturing solution conditions in the presence of either 50 or 200 mM NaCl. These salt concentrations positioned the monomers well inside either the monomeric or oligomeric fibril assembly pathways described above. At both salt concentrations lysozyme became marginally more disordered when raising the temperature from 37 to 50°C (data not shown). Yet, neither the secondary (see Fig. 4 A) nor the tertiary structure (data not shown) of lysozyme showed any discernable differences between the two salt concentrations. Similarly the midpoint temperature for lysozyme denaturation (55°C) was unaffected by changes in NaCl concentrations (Fig. 4B). The steep sigmoidal shape of the temperature profile remained invariant with changes in salt concentration, as well. Hence, there were no signs that increased NaCl induced additional folding intermediates such as a molten globule state [33]. Instead, lysozyme denaturation remained a two-stage transition between a native and a denatured state [34].

### Intermolecular Interactions among Partially Denatured Monomers

The above observations imply that there are no pronounced structural changes in the secondary or tertiary structure of partially denatured monomers that would account for the salt-induced transitions in aggregation behavior. There are several indications however that the transition might be related to the effects of salt-screening on the charge interactions among the partially denatured lysozyme monomers and the aggregates they form. First of all, under the acidic solution conditions used in our experiments lysozyme carries a substantial net charge of +15 e [21], [22]. Furthermore, oligomeric aggregates with compact geometries only begin to form as salt concentration increases while the monomeric filaments nucleating at low salt concentrations all assume a highly extended conformation. The transition from an extended to compact geometry for the initial intermediate is consistent with the idea that repulsive charge interactions would suppress small compact intermediates until salt screening helps to overcome the energy cost imposed by long-range charge repulsion. Finally, it is well established that the intermolecular interactions of lysozyme undergo pronounced changes in response to salt-screening [20], [35], [36], [37].

**Figure 5:**
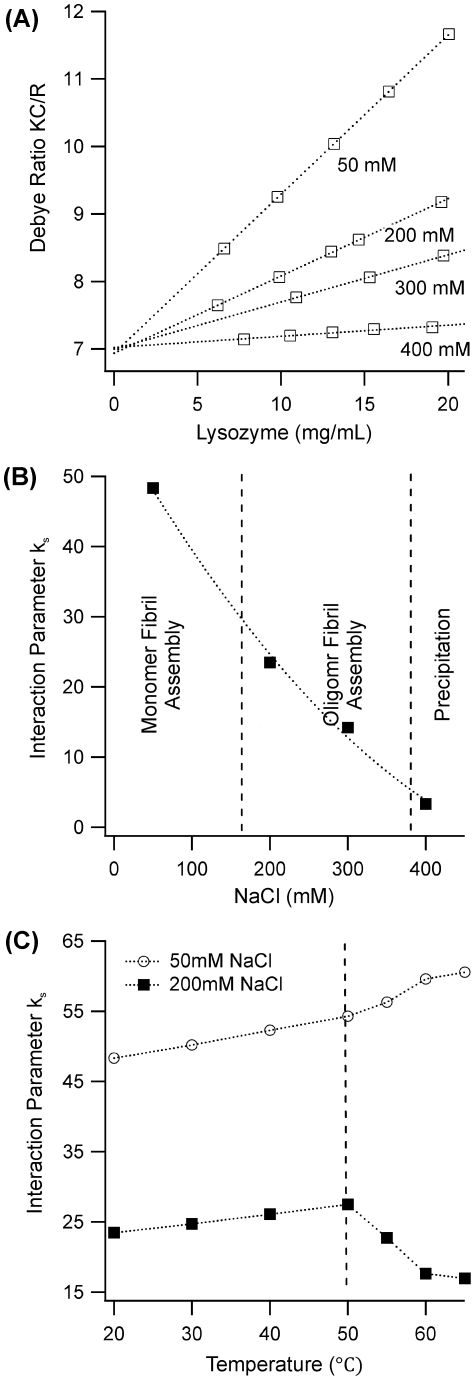
Net Interactions among Native and Denatured Lysozyme Monomers. (**A**) Debye plot of the static light scattering intensity (KC/R) *vs.* lysozyme concentration C at T = 20°C. The positive slope of these curves indicates that the interactions among the lysozyme monomers are predominately repulsive. This repulsion becomes screened out once NaCl concentrations reach about 400 mM. (**B**) Plot of the static interaction parameter k_s_ (which is proportional to the slope of KCp/R *vs.* Cp) *vs.* salt concentration for the Data in A. The dotted line is a guide to the eye indicating how repulsion decreases with increasing salt concentration. The two dashed vertical lines mark the switch of lysozyme aggregation from monomeric (MF) to oligomeric fibril (OF) assembly and, eventually, precipitate formation (P). (**C**) Change in net interactions as lysozyme monomers undergo thermal denaturation in the presence of 50 mM (○) and 200 mM (▪) NaCl. The vertical dashed line indicates the onset of thermal denaturation at 50°C. Note that, the prevailing intermolecular interactions remain repulsive (positive K_s_ values) even after thermal denaturation. At the same time, denaturation at 50 mM NaCl makes lysozyme slightly more repulsive while the monomers become less repulsive following denaturation at 200 mM NaCl.

We used static light scattering (SLS) to determine the character of the intermolecular interactions among lysozyme monomers undergoing amyloid formation. Specifically, we investigated net lysozyme interactions at four different salt concentrations (50, 200, 300 and 400 mM NaCl) located in the three different aggregation regimes (monomeric *vs.* oligomeric fibril assembly *vs.* precipitation), respectively. Near the denaturation temperature, solutions at 300 and 400 mM rapidly became unstable toward aggregation. Hence, we initially measured interactions at 20°C. Fig. 5 A shows the Debye-plot of the scattering parameter KC/R *vs.* lysozyme concentration at each salt concentration. The slope of KC/R for lysozyme is positive at low and intermediate salt concentrations and reaches almost zero near 400 mM NaCl. Positive slopes at low salt concentrations indicate the prevalence of charge repulsion among the monomers. As charge repulsion is increasingly screened out by the double layer formed by salt ions, short-range attractive interactions begin to prevail. As shown in Fig. 5B, the changes in the intermolecular interaction parameter k_s_ can be readily extrapolated and indicate that the sum of short-range attraction and long-range charge repulsion begin to cancel each other around 400 mM NaCl.

It is not obvious *a priori* that the intermolecular interactions at 20°C among folded monomers are representative of the interactions among the partially denatured monomers near 50°C. We therefore investigated how temperature-induced denaturation of lysozyme altered its intermolecular interactions. These measurements were limited to lower salt concentrations where charge repulsion prevented rapid aggregation. In addition, we took special precautions to eliminate contamination of interaction measurements from aggregate formation (see Materials and Methods). Intriguingly denaturation in the monomeric *vs.* the oligomeric aggregation regime changed lysozyme's intermolecular interactions in opposite direction (Fig. 5C). In the monomeric aggregation regime ([NaCl] = 50 mM), net interactions became slightly more repulsive (slope increases) upon reaching the denaturation threshold of 50°C. This increase is probably indicative of the increased volume the monomers take up upon denaturation, which is equivalent to an enhanced hard-core repulsion. In contrast, net intermolecular interactions in the oligomeric aggregation regime ([NaCl] = 200 mM) became more attractive upon denaturation. Hence, at intermediate salt concentrations denatured monomers can sample some of their short-range attractive interactions among their exposed hydrophobic cores. Overall, though, amyloid fibril formation occurs under conditions in which charge repulsion dominates the intermolecular interactions among the denatured monomers.

It is important to remember that the net interaction parameter measured here quantifies *net two-body* interactions. Partially denatured monomers will sample a much broader range of conformations than their native counterparts. As a result, any specific pair of denatured monomers will experience a range of different values for their mutual interactions, depending on their respective conformations and the amount of the hydrophobic core these conformations expose. *Two-body* repulsion further implies that that the repulsive contribution from lysozyme's net charge is unlikely to be overcome by only *two* interacting monomers. Instead, aggregation will require multiple monomers to coalesce, thereby increasing their attractive contact regions. This provides a natural explanation why at least eight monomers are required to stabilize oligomeric intermediates, which is the smallest amyloidogenic intermediate in our system.

### Salt-mediated Effects on Amyloid Assembly: Charge-Screening vs. Ion-Specific Effects on Protein Interactions?

**Figure 6:**
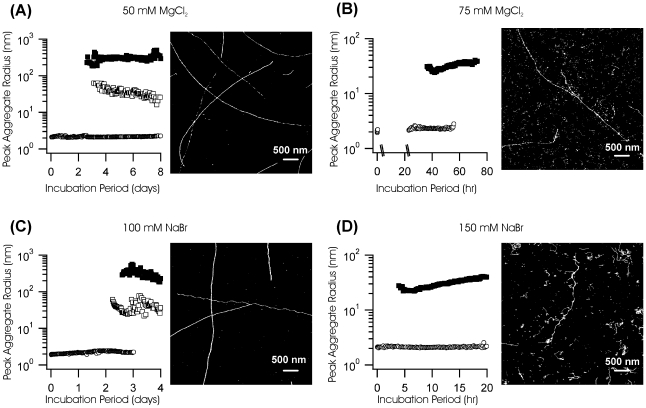
Lysozyme Fibril Growth in the Presence of MgCl_2_ vs NaBr. DLS nucleation and growth kinetics (left panel) and corresponding AFM images of late-stage aggregates (right panel) for lysozyme amyloid fibrils grown in (**A**) 50 mM MgCl_2_, (**B**) 75 mM MgCl_2_ (**C**) 100 mM NaBr and (**D**) 150 mM NaBr.

The above static scattering data indicate that intermolecular protein interactions are strongly modulated by the concentration of sodium chloride. However, there are multiple ways salt ions can modulate protein interactions. Besides charge screening via the Debye-Hückel double-layer of diffusive ions, ion-specific absorption onto the protein or ion-specific changes to solvent-mediated hydrophobic/hydrophilic interactions could underlie the interaction changes. Using different salt ions carrying different net charges, we evaluated the relative importance of diffusive salt screening *vs.* ion-specific changes to protein interactions. The extent of diffusive charge screening is solely determined by the ionic strength I of the solutions, given by


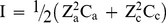
(1)

where *C_a_, C_c_* are the molar concentrations of anions and cations and *Z_a,_ Z_c_* are their respective charge numbers [38]. If salt-mediated charge screening is the dominant mechanism for the switch from monomeric to oligomeric fibril assembly, this transition should occur at comparable ionic strengths I for different salts. We repeated lysozyme aggregation studies using either NaBr or MgCl_2_. This selection altered either the co-ion (Mg^2+^ vs. Na^+^) or counter-ion (Cl^−^ *vs.* Br^−^) and included a divalent ion (Mg^2+^). For both salts, though, we observed the same transition from monomeric fibril assembly at low ionic strength to oligomeric fibril assembly at intermediate ionic strength (see Fig. 6). More specifically, the transition occurred near 150 mM for NaCl and NaBr while it shifted to between 50 and 75 mM for the divalent Mg^2+^ ion. Hence, non-specific charge screening by salt ions represents the dominant mechanism driving the transition from monomeric to oligomeric fibril assembly.

## Discussion

Together, the above data indicate that aggregation of amyloidogenic lysozyme falls into two broad categories: up to modest salt concentrations (<350 mM) lysozyme assembles into amyloid fibers while, at elevated salt concentration, disordered protein precipitation sets in. Within the regime of moderate salt concentrations, amyloid fibril self-assembly itself abruptly switches from a monomeric to an oligomeric assembly pathway. Monomeric assembly prevails at low salt concentrations (<150 mM NaCl) and involves the nucleation of two filament populations of different length (MF-L and MF-S). Their physical characteristics imply that these filaments are linear assemblies of monomers. Monomeric filaments eventually cross-link into mature fibrils composed of three monomeric filaments (MF) per fibril. No compact, oligomeric intermediates are detected in this regime. For intermediate salt concentrations (150 mM>[NaCl]<350 mM), compact oligomeric intermediates are the basic building block for all subsequent fiber assemblies. Compact, globular oligomers begin to form without discernable lag period. Eventually, oligomeric filaments (OF) nucleate, which themselves grow in length and become increasingly curvilinear. As indicated by the doubling in cross-sectional area, double-stranded mature fibrils are formed by cross-assembly of two oligomeric filaments (OF).

The presence of distinct amyloid assembly pathways for lysozyme together with the detailed morphological characterization of all intermediates within each pathway, to our knowledge, has not been described in this system before. Earlier *in vitro* studies did use pH = 2, but typically very low ionic strength. In addition, morphological characterizations were performed at the late stages of aggregation, long past the formation of intermediates we report here. [27], [29], [39], [40]. Keeping these limitations in mind, these earlier reports observed long, very rigid fibrils under low-salt conditions at pH = 2, consistent with the low-salt monomeric filament pathway in our experiments.

The observations of distinct pathways with distinct intermediates, however, does resemble those made with β_2_-microglobulin, the protein underlying dialysis-related amyloidosis [12]. Specifically, our monomeric filaments share many features with "long-straight" fibrils while oligomeric filaments resemble "rod-like" or "worm-like" β_2_-microglobulin fibrils. The ability to interconvert rod-like and worm-like β_2_-microglobulin fibrils is consistent with the interpretation that the former represent the same species of oligomeric filaments at an earlier stage of their growth process. While our experiments were not specifically designed to address the question whether monomeric and oligomeric assembly pathways compete with one another or represent distinct assembly pathways, our data support the latter interpretation. First, mature fibrils assembled under either "monomeric" or "oligomeric" growth conditions show clearly distinct physical characteristics (Fig. 1C and table 1). Ideally one would like to determine the structure of composite fibrils, and the specific number of filaments they contain, using high-resolution structural information. Yet, the integer increments in the cross-sectional areas of mature fibrils compared to their precursors (Fig. 1C) imply that mature fibrils emerge *via* cross-assembly of either three monomeric filaments (monomeric assembly pathway) or two oligomeric filaments (oligomeric assembly pathway). Hence, we suppose that monomeric *vs.* oligomeric growth conditions represent two distinct pathways that, under our conditions, do not coexist.

### Net Protein Interactions as Regulator of Fibril Assembly vs. Precipitation

The switch from ordered fibril assembly at low and intermediate salt concentrations to disordered precipitation at elevated salt concentrations (Fig. 1 *vs.* 3) closely correlates with the salt-induced transition from net repulsion to net attraction among lysozyme monomers (Fig. 5B). This correlation might appear counterintuitive, at first. It is well-established that *modest attractive* interactions are a necessary precondition for protein crystallization of native proteins, while precipitation sets in as net attraction exceeds a threshold value [16], [19], [20], [36]. At the same time, the low-salt, low pH values used during amyloid fibril growth have been shown to result in net repulsion, at least while lysozyme remains below the unfolding transition [20], [35], [37]. Our data in Fig. 5C confirm that net interactions remain repulsive even after lysozyme has undergone denaturation. This apparent contradiction can be readily resolved by considering the distinctly different role of protein charge interactions during amyloid fibril assembly vs. protein crystallization. First, as long as solution temperature was kept below the denaturation temperature, lysozyme monomers did indeed never aggregate since their mutual interactions are repulsive (Fig. 5C). Raising the solution temperature to partially denature lysozyme was a necessary condition for fibril assembly. Such partial denaturation as a pre-condition for amyloid formation of native proteins has been well documented [41]. However, partial denaturation alone does not turn the prevailing net repulsion into attraction (Fig. 5C). Instead, net charge repulsion prevents partially denatured monomers from forming intermolecular cross-links, unless their conformations permit them to establish beta-sheet bonds (low salt) or micelle-like structures sharing hydrophobic interactions. Once net repulsion is abolished, any of the partially denatured monomers can begin associating and random precipitation sets in. Hence, we consider charge repulsion to play the role of a “gate keeper” for those conformations of partially denatured monomers that can form energetically sufficiently favorable, ordered precursors for amyloid formation.

### Charge Repulsion as Switch between Monomeric and Oligomeric Assembly Pathways

**Figure 7:**
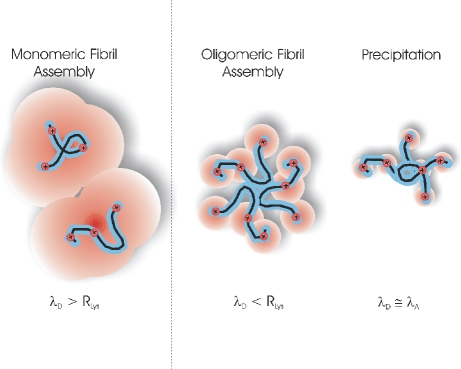
Effects of Salt-mediated Charge Screening on Denatured Monomers. The schematic indicates how the spatial extent (Debye screening length λ_D_) of salt-mediated charge screening changes the character of the net interactions among denatured monomers and favors the formation of different aggregate geometries. The black curvy line represents the protein backbone while the blue perimeter symbolizes the short-range attractive protein interactions (hydrophobic, dipole-dipole, hydrogen bonding). Individual charged residues are represented by small positive spheres, and the extent of charge screening mediated by the salt ions is indicated as a red cloud surrounding the charge residues. At low salt concentrations, (monomeric assembly pathway) individual charges on the same monomer strongly repel each other and those on neighboring monomers. Only those few conformations of denatured monomers that can form intermolecular bonds similar to those in the native monomer are aggregation competent. In addition, charge repulsion among monomers will favor extended, polymeric structures for intermediates since that will separate the monomer charges from each other while preserving sufficient intermolecular contacts. When salt screening reduces λ_D_ below the separation of charged residues (oligomeric assembly pathway) along the monomer backbone, charge repulsion within a given monomer and, concurrently, among several aggregated monomers is significantly reduced. This favors the formation of more compact (oligomeric) aggregate assemblies and requires fewer monomers to share their hydrophobic cores to overcome the residual charge repulsion and loss in configurational entropy. Finally, when λ_D_ becomes comparable in range to the attractive interactions, the charge restrictions on "suitable" aggregate morphologies and favorable monomer conformation fall by the wayside and the denatured monomers precipitate randomly out of solution.

So far, we have argued that the transition from net repulsive to attractive protein interactions provides a natural explanation for the salt-induced transition from fibril assembly to precipitation. We further contend that charge interactions, which remain repulsive under fibril growth conditions, also trigger the transition from monomeric to oligomeric fibril assembly pathways. We noted that the transition from monomeric to oligomeric fibril assembly occurs at comparable ionic strengths, independent of salt identity (Fig. 6). Debye-Hückel theory indicates that salt screening is associated with an intrinsic length scale λ_D_

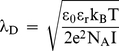
(2)


where ε_0_ and ε_r_ are the vacuum permittivity and dielectric constants of the medium, k_B_T is the thermal energy and *I* is the ionic strength. λ_D_ represents the distance over which salt ions screen out the long-range charge repulsion among two charged proteins. To a first approximation, it also sets the length scale over which individual charge residues *within* a given protein polymer become screened from one another. Using the ionic strength of 150 mM for the transition from monomeric to oligomeric assembly and ε_r_ = 70 for water at T = 50°C, the corresponding screening length λ_D_ becomes 0.78 nm. This is a factor of 2–3 below the hydrodynamic radius *R_h_* = 1.9 nm for lysozyme [42]. Hence, the transition occurs as the repulsive interactions among individual charged residues on the same monomer become screened (Fig. 7).

This transition from charge repulsion among multiple monomers to partial screening of charges within a single monomer puts different constraints on both the geometry of intermediate aggregates and their rate of formation. First charge repulsion strongly favors only those intermediates that can form sufficiently large numbers of favorable *intermolecular* contacts, similar to those stabilizing the structure of the native monomers. At low salt concentrations in particular, the large range of charge repulsion favors aggregate structures that are tightly bound while spreading out the charges along the aggregate, thereby reducing the repulsive electrostatic contributions to the aggregate's free energy. This charge constraint on aggregate morphology favors linear aggregate geometries. As the Debye screening length drops below the typical separation of charged groups within a monomer, compact (oligomeric) intermediates become more favorable since their geometry provides more opportunities for intermolecular contacts across multiple monomeric units. In addition, the entropic constraints are likely to be relaxed since a larger number of denatured conformations can participate in sharing their hydrophobic cores. Such simultaneous relaxation of both energetic and entropic constraints on aggregate geometries provides a compelling reason for the sharp transition in aggregation pathways.

### Model for the Effects of Charge Interactions on Amyloid Fibril Assembly

**Figure 8:**
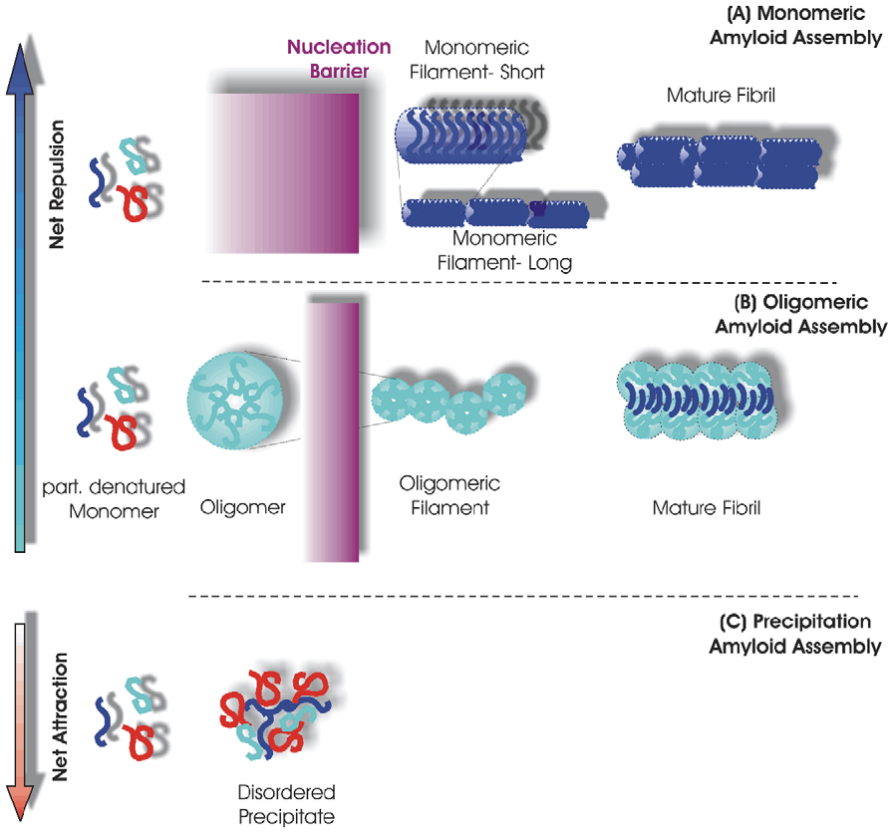
Schematic of Lysozyme Amyloid Assembly as Function of Net Intermolecular Interactions. On the left side, the schematic indicates a collection of partially denatured monomers in different conformations. The relative distribution of denatured conformations will vary both with solution temperature and with the intramolecular interactions, which are affected by salt screening, as well. At the lowest salt concentration, charge repulsion suppresses the formation of compact oligomeric intermediates with their high local concentration of charge. Instead, the monomers have to form extended "short" monomeric filaments that spread out the net charge and which are held together by strong hydrogen cross-links (β-sheets). This process has a much higher nucleation barrier since many more monomers have to condense into the nucleus and the corresponding extended conformations of the denatured monomers are bound to be less frequently populated. Short monomeric filaments can assembly head-to-head into long monomeric filaments. These, in turn, can form mature fibrils via cross-assembly of three monomeric filaments. As salt concentration is increased (middle third of plot), lysozyme interactions become less repulsive. In this regime, monomers can assemble into oligomeric intermediates if they share their hydrophobic cores. This happens without apparent nucleation barrier. The nucleation step here is the formation of oligomeric filaments with, we suppose, partially overlapping core structures. These oligomeric filaments can form cross-links and restructure to form mature fibrils. At elevated salt concentrations (bottom third of plot), charge repulsion among the lysozyme monomers will be screened out and net intermolecular interactions become exclusively attractive. In this regime, monomers will undergo diffusion-limited aggregation and form random precipitates.

The above considerations lead us to propose the following model for self-assembly of lysozyme under amyloidogenic solution conditions (Fig. 8). At low salt concentrations, only long, extended monomeric filaments can overcome the constraints imposed by charge repulsion on bond strength (β-sheets) and aggregate geometry. These monomeric filaments eventually cross-assemble into thicker mature fibrils with three filaments linking up to form a fibril strand. As charge repulsion becomes sufficiently screened out at intermediate salt concentrations, the formation of compact oligomeric intermediates becomes both energetically and entropically more favorable. These oligomers form immediately and, as their concentration increases, nucleate and grow into oligomeric filaments with a pronounced curvilinear geometry. Oligomeric filaments then cross-assemble into mature fibrils composed of two oligomeric filaments per fiber. These mature fibrils are clearly distinct from those emerging at low salt concentrations. Finally, as screening length decreases further, short-range attractive interactions balance or overcome charge repulsion. At that point, constraints on aggregate morphology and favorable conformations among the binding partners are eliminated and lysozyme monomers assemble into random precipitates.

This model provides a compelling rationale for the transition among different amyloid assembly pathways and helps to rationalize the geometry of their intermediates. It explains why and when partially denatured monomers precipitate instead of forming ordered fibril assemblies. Equally intriguing, these considerations highlight that charge interactions play distinctly different roles during crystallization or liquid-liquid phase separation of native, folded proteins *vs.* their role during self-assembly of fibrillar structure from partially denatured or disordered proteins.

## Materials and Methods

### Protein and Chemicals

For all experiments, 2× recrystallized, dialyzed and lyophilized lysozyme from Worthington Biochemicals (Lakewood, NJ) was used. All other chemicals were from Fisher Scientific (Pittsburgh, PA), and were reagent grade or better. 18 MΩ RO purified water (Barnstead E-pure, Dubuque, IA) was used throughout.

### Preparation of HEWL Samples

Solutions of lysozyme were prepared by dissolving lyophilized lysozyme at twice its final concentration in 25 mM KH_2_PO_4_ pH 2 buffer, and mixing it 1∶1 with a NaCl/25 mM KH_2_PO_4_ buffer solution, also at twice its final NaCl concentration. Before mixing, lysozyme solutions were warmed to 45°C to remove any preformed clusters. All samples were filtered consecutively through a 220-nm and a 20-nm pore size syringe filter. Actual lysozyme concentrations were determined from uv absorption measured at *λ* = 280 nm (a_280_ = 2.64 mL mg^−1^ cm^−1^).

### DLS Kinetics during Amyloid Aggregation

DLS measurements were performed with a Zetasizer Nano S (Malvern Instruments, Worchestershire, UK) containing a 4 mW He-Ne laser (*λ* = 633 nm) with built-in temperature control for sample cuvettes. After thermal equilibration of the samples (typically 10 min), autocorrelation functions were collected every 10 min, using acquisition times of 180 s. Autocorrelation functions were converted into particle-size distributions, using the “narrow modes” or “general purpose” algorithms provided with the Zetasizer Nano S.

### SLS Measurements of Lysozyme's Net Intermolecular Interactions

SLS measurements of lysozyme's interaction parameters were performed under two different set of conditions. Below the denaturation temperature of 50°C, measurements could be completed with NaCl concentrations up to 400 mM without signs of aggregate formation (Fig. 5A). Conversely, measurements above the denaturing temperature (Fig. 5C) could only be performed up to NaCl concentrations of 200 mM. In the later case, the presence of a nucleation barrier suppressed the onset of any significant aggregation for at least several hours, i.e. long after the completion of our SLS measurements. In all cases, the reported data are derived from averaging 5–10 intensity values sampled for 1 minute each. Sequential measurements were scrutinized for any signs of systematic, time-dependent increases suggestive of aggregation. Since the SLS measurements were performed with the Malvern DLS unit, we simultaneously obtained correlation functions. Only samples with polydispersities of their correlation functions below 0.07 were included in our measurements. Finally, the y-axis intercepts for all measurement (see Fig. 5A) yielded the inverse of lysozyme's monomeric molecular weight of 14.3 kD. These precautions exclude noticeable contamination of the data by the presence of equilibrium or non-equilibrium aggregation.

### Static Light Scattering Analysis and Direct Protein Interactions

For static light scattering the excess scattering from the solution due to lysozyme *(I_tot_ – I_sol_)* is compared against the scattering from a known standard like toluene and quantified as the Rayleigh ratio, *R*_*θ*_

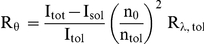



where *I_tot_, I_sol_, I_tol_* are the measured scattering intensity of the protein solution, the salt/buffer background and the toluene standard, respectively. The ratio *(n_o_/n_tol_)^2^* accounts for the differences in observed scattering volume in the two solvents and *R_λ,tol_* is the Rayleigh ratio for toluene at the measurement wavelength. For *λ* = 633 nm, we used a value of *R_λ,tol_* = 13.5×10^−6^ cm^−1^. For typical stgciqengths of protein interaction, the normalized Rayleigh ratio increases linearly with protein volume fraction and can be presented by the lowest-order virial expansion
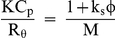



where *M* is the molecular weight of the protein, *k_s_* is the direct interaction parameter, and *Φ* is the protein volume fraction. The optical constant *K* is given by *K* = *(2 π ^2^ n_o_^2^*/*N_A_ λ_o_^4^) (dn_o_ /dC_p_) ^2^* where *N_A_* is Avogadro's number, *λ_o_* is the wavelength of incident light, and the differential refractive index increment for lysozyme at our wavelength is *(dn_o_ /dC_p_)* = 0.185 ml/g. This relationship was used to obtain the static interaction parameters *k_s_* for lysozyme under different solution conditions (see Fig. 5).

### Circular Dichroism (CD)

CD measurements were carried out on an Aviv Biomedical Circular Dichroism Spectrometer, Model 215 with a temperature controlled cell holder. For far-UV wavelength measurements, the protein concentration was 30 µM and the quartz cell path length was 1 mm. The wavelength scans were measured from 190–260 nm with a step size of 1 nm and an averaging time of 10 seconds per step. Each measurement was an average of three scans with a temperature equilibration time of five minutes. For the temperature denaturation scans, the signal was monitored at 222 nm with a temperature scan rate of 1°C per minute. The temperature step size was either 5°C or 2°C with a temperature equilibration time of 5 minutes and a 10 second averaging time per step.

### AFM Characterization of Amyloid Aggregates

Amyloid samples were imaged in air with a MFP-3D atomic-force microscope (Asylum Research, Santa Barbara, CA) using NSC36/NoAl (Mikromasch, San Jose, CA) or PPP-FMR (Nanosensor, Neuchatel, Switzerland) silicon tips with nominal tip radii of 10 nm and 7 nm, respectively. The cantilever was driven at 60–70 kHz in alternating current mode and a scan rate of 0.5 Hz, acquiring images at 1024×1024-pixel resolution. Raw image data were corrected for image bow and slope. As previosly described [28], AFM tips were calibrated to correct for the dilation in the apparent particle width by imaging 5-nm gold colloid standards (GC5, BBI International).

During DLS measurements of amyloid fibrillogenesis, aliquots of solution were taken from the DLS cuvette for subsequent AFM imaging. Aliquots were diluted 100-fold for 175 mM NaCl solutions and 20-fold for 50 mM NaCl solutions. Typically, 75 µL of the solution was deposited onto freshly cleaved mica, rinsed with deionized water, and dried with dry nitrogen. For 175 mM NaCl solutions, all aggregated samples were deposited onto mica for 5 minutes, while monomers were deposited for 15 minutes. For 50 mM NaCl solutions, all samples were deposited for 15 minutes except the mature fibrils which were deposited for 5 minutes.
